# Experimental study of thermal comfort on stab resistant body armor

**DOI:** 10.1186/s40064-016-2432-x

**Published:** 2016-07-26

**Authors:** Tingchao Ji, Xinming Qian, Mengqi Yuan, Jinhui Jiang

**Affiliations:** State Key Laboratory of Explosion Science and Technology, Beijing Institute of Technology, Beijing, China

**Keywords:** Thermal comfort, Exercise intensity, Exercise sequence, Core temperature, Skin temperature, Subjective judgements

## Abstract

**Purpose:**

This research aims to investigate the impacts of exercise intensity and sequence on human physiology parameters and subjective thermal sensation when wearing stab resistant body armor under daily working conditions in China [26 and 31 °C, 45–50 % relative humidity (RH)], and to investigate on the relationship between subjective judgments and objective parameters.

**Methods:**

Eight male volunteers were recruited to complete 3 terms of exercises with different velocity set on treadmill for 90 min at 26 °C and 31 °C, 45–50 % RH. In Exercise 1 volunteers were seated during the test. In Exercise 2, volunteers walked with the velocity of 3 km/h in the first 45 min and 6 km/h in the left 45 min. In Exercise 3, volunteers walked with the velocity of 6 km/h in the first 45 min and 3 km/h in the left 45 min. The body core temperature, skin temperature and subjective judgments were recorded during the whole process. Analysis of variance was performed among all the tests.

**Results:**

Individual discrepancy of Exercise 1 is larger than that of Exercise 2 and 3. On the premise of the same walking distance and environmental conditions, core temperature in Exercise 3 is about 0.2 °C lower than that in Exercise 2 in the end; and with the velocity decrease from 6 km/h to 3 km/h in the end, thermal tolerance of Exercise 3 is about 1 degree lower than that in Exercise 2. Skin temperatures of human trunk were at least 1 °C higher than that of limbs.

**Conclusions:**

Activity narrows the individual discrepancy on core temperature. Within experimental conditions, decreasing of intensity at last stage makes the core temperature lower and the whole process much tolerable. The core temperature is more sensitive to the external disturbance on the balance of the whole body, and it can reflect the subjective thermal sensation and physical exertion.

## Background

In recent years with terrorist incidents continuing to occur, protective clothing has become of importance, and is required to be worn for occupational safety and healthy purposes, i.e., for law enforcement internationally (Jimenez et al. 2012; Majumdar et al. 1997). It is generally accepted that the officers has faced a greater threat from knives and other sharp utensils than they do from bullets, since the usage of gun and other firearms are under controls worldwide (Croft and Longhurst 2007a, b). Thus the stab resistant body armor (SRBA) is now a standard item of equipment for police officers (Dempsey et al. 2013; Ian et al. 2005), which can buffer the damage from edged weapon to the wearer, therefore protect their lives.

At present, the SRBA is divided into 3 main categories according to the material of its protection layers: hard, half hard and soft (Ding et al. 2011). Hard SRBA is the first version of the SRBA, of which the protection layer is made from rigid materials (i.e. metal, ceramic, composite plate). Although excellent protection performance was presented, hard SRBA has huge limitations on the flexible action and wearing comfort (Decker et al. 2007). Half hard SRBA contains both metal layer of metal wires (i.e. metal ring mesh) or metal cords (i.e. titanium foil) and textile layer, with improved flexibility (Bourgois et al. 2001; Decker et al. 2007). The core layer of soft SRBA is the high performance fiber fabric such as ultrahigh molecular modulus polyethylene (UHWMPE), aramids, polybenzoxazole, and polyamide (Flambard and Polo 2004; Lin et al. 2011), which has become the focus of the protection products research (Ding et al. 2011).

The SRBA aims to provide protection from stabbing and spread or expend the most energy of the damage (Croft and Longhurst 2007a, 2007b; Ding et al. 2011). To guarantee the protection performance, SRBA usually have a low permeability, poor thermal characteristics in addition to the heavy weight [usually 3–4 kg (Davis and Bishop 2013; Dempsey et al. 2013, 2014)], thus the wearer heat load was increased. Human body thermal balance can be disrupted by a variety of factors, including exercise intensity, clothing, ambient temperature, humidity and so on (Davis and Bishop 2013). An essential requirement for continued normal body function is that the deep body temperature should be maintained within 37 ± 1 °C (Epstein and Moran 2006). When the officer discharging duties wearing the SRBA, the body core temperature will constantly increase, resulting low human thermal comfort due to: the heat generated from the body through sweat evaporation is difficult to discharge, and heavy workloads will lead to continuous heat accumulation in the body. When the core temperature exceeds 38 °C, there are well-documented physiological effects on human body (Lundgren et al. 2014), such as heat stress and dehydration, making it hard to work productively, especially physically and cognitively (Bennett et al. 2010). Paddy. C et al. (Dempsey et al. 2013, 2014) investigated the effect of added load and exercise intensities on jump and landing performance and the subject mobility wearing SRBA. The results showed that the mean performance decreases ranged from 13 to 42 % while loaded. Chantal. J et al. (Jimenez et al. 2012) confirmed the deleterious effect of wearing protective clothing on physiological tolerance due to the microenvironment created inside the clothing.

A major factor contributing to the human thermal comfort is the heat transfer between the human body and the environment (Starr et al. 2015), which is reflected by human temperature change. Obtaining human physiological parameter and subjective evaluation results when wearing the SRBA is an essential approach to minimize the heat strain and optimize the comfort of the body armor (Levine et al. 1998). Research on human thermal comfort has been conducted mainly focused on the impact of high ambient temperature (>35 °C) and heavy weight protective clothing (>10 kg) (Huiju et al. 2013; Majumdar et al. 1997), such as firefighting clothing or ballistic body armor (Caldwell et al. 2011; Lee et al. 2013). Generally, SRBA is much lighter than the ballistic body armor, which is about 3 kg in weight in this research. And the average daily working temperature in China of summer is about 30 °C. The purpose of this research is to investigate the impact of exercise intensity on human physiology parameters and subjective thermal sensation when wearing SRBA under daily working conditions in China (26 and 31 °C, 45–50 % relative humidity), and the relationship between the subjective judgement and the objective temperature change was analyzed.

## Methods

### Subjects

Two sets of experiments were designed and performed. One set is conducted at 26 °C, 45–55 % RH, the other is conducted at 31 °C, 45–55 % RH. Eight undergraduated healthy male volunteers were recruited in total from Beijing Institute of Technology, China. Basic physiological information of the subjects is shown in Table 1. All tests were conducted from April to May 2015. Subjects had been told the procedures and relative potential risks of the experiments prior to providing written informed consent. Also, they were required to work and rest regularly, eat healthy, drink more water, and not to take energetic exercise.

**Table 1:** Characteristic of subjects in each sets ($\bar{x} \pm SD$)

Ambient condition	Age (years)	height (cm)	weight (Kg)	BMI
26 °C (45–55 % RH)	23.25 ± 0.5	179.00 ± 6.58	69.55 ± 8.12	21.64 ± 1.15
31 °C (45–55 % RH)	22.75 ± 0.5	178.25 ± 6.90	71.37 ± 7.86	22.41 ± 1.42

### Materials and apparatus

The experiment was conducted in a climate chamber, of which the wall is made of polyethylene and structured steel, so that the temperature and humidity could be individually controlled at a certain range. The size of the climate chamber is $2 {\text{m}} \times 2 {\text{m}} \times 2 {\text{m}}$ (${\text{length}} \times {\text{width}} \times {\text{height}}$).

**Fig. 1:**
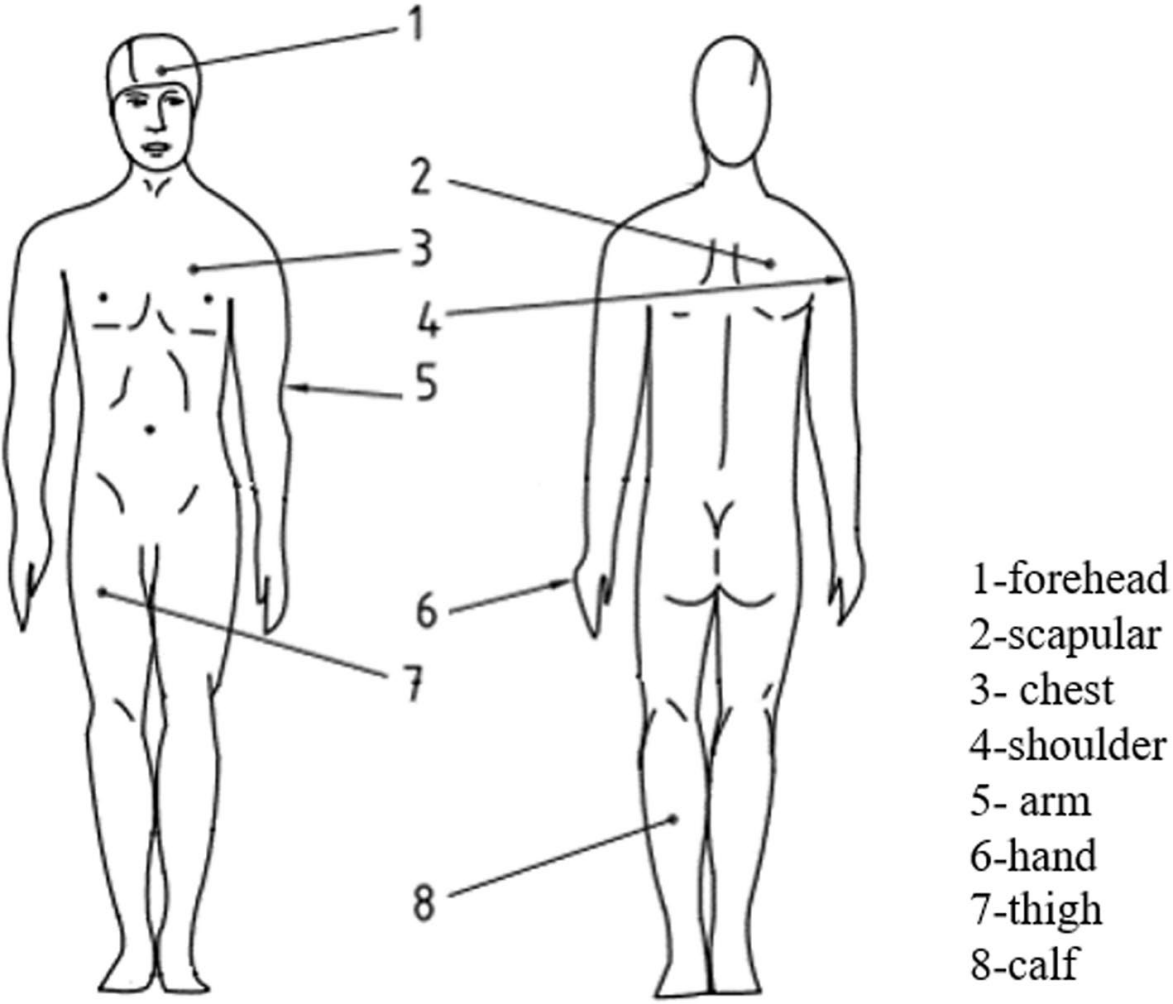
Location of measuring sites

Ambient temperature and relative humidity in the climate chamber were monitored. The relative humidity was controlled at the range of 45–55 %. Eight thermocouples (PT100, China; accuracy: ±0.1 °C) were fixed on the subject skin to collect local skin temperatures, sticking by surgical breathable tape. The skin temperature was measured at 8 points (ISO 2004; Thornley et al. 2003) over the whole body. The location of measuring sites is shown in Fig. 1, which including the forehead, right scapular, left upper chest, right shoulder [described as arm in upper location in the European Standard EN ISO 9886:2004 (ISO 2004)], left arm in lower location, left hand, right anterior thigh and left calf. Skin temperature data was recorded by using a data logger (XSR90, China; recording time interval: 0.1 s) in every second. Rectal temperature was measured by inserting a rectal thermometer (OMRON, US; accuracy: ±0.2 °C) through the anus. Exercise intensity was changeable and controlled using a treadmill.

A stab resistant body armor (FCF-F-ZT03, China), which protection layer is made from UHWMPE was used in this experiment, which is 3.5 kg in weight and 0.3 m^2^ of protective area. The SRBA is detachable and sleeveless, and meets the stab resistant body armor standard of China (Standard 2008). Subjects were required to wear a quilted T-shirt under the SRBA.

The ANOVA for the six groups (three under each ambient condition) was performed using the statistical software package SPSS 20.0 for Windows.

### Experimental procedures

The experimental temperatures were set at 26 and 31 °C respectively for each session.

Three trails were performed under each environmental condition. According to the classification of metabolic rate (Ergonomics of The Thermal Environment—Determination Of Metabolic Rate), three classes of metabolic rate were chosen: resting (sitting at ease, about 65 W/m^2^), moderate (3 km/h, about 142 W/m^2^), high (6 km/h, about 220 W/m^2^). Within each trail, rectal temperature was measured every 5 min, and local skin temperature was recorded every 15 s. The thermal sensation questionnaire was filled every 15 min.

Exercise 1 is the pre-task trail, during which subjects were sedentary in the climate chamber for 90 min. Exercise 2 and 3 were divided into 6 cycles. In each cycle, subjects walked on a zero-grade treadmill for 12 min followed by a 3 min rest. During the first 45 min of Exercise 2, the walking velocity was set as 3 km/h, and in the rest 45 min the velocity was set as 6 km/h. Conversely, the velocity of Exercise 3 in the first 45 min was 6 km/h and the velocity in the left 45 min was 3 km/h, as shown in Table 2.

**Table 2:** Velocity of each exercise during the whole process

Time procedure (min)	0–15		15–30		30–45		45–60		60–75		75–90	
Time interval (min)	12	3	12	3	12	3	12	3	12	3	12	3
Velocity (km/h)												
Exercise 1	0	0	0	0	0	0	0	0	0	0	0	0
Exercise 2	3	0	3	0	3	0	6	0	6	0	6	0
Exercise 3	6	0	6	0	6	0	3	0	3	0	3	0

### Subjective judgment

**Fig. 2:**
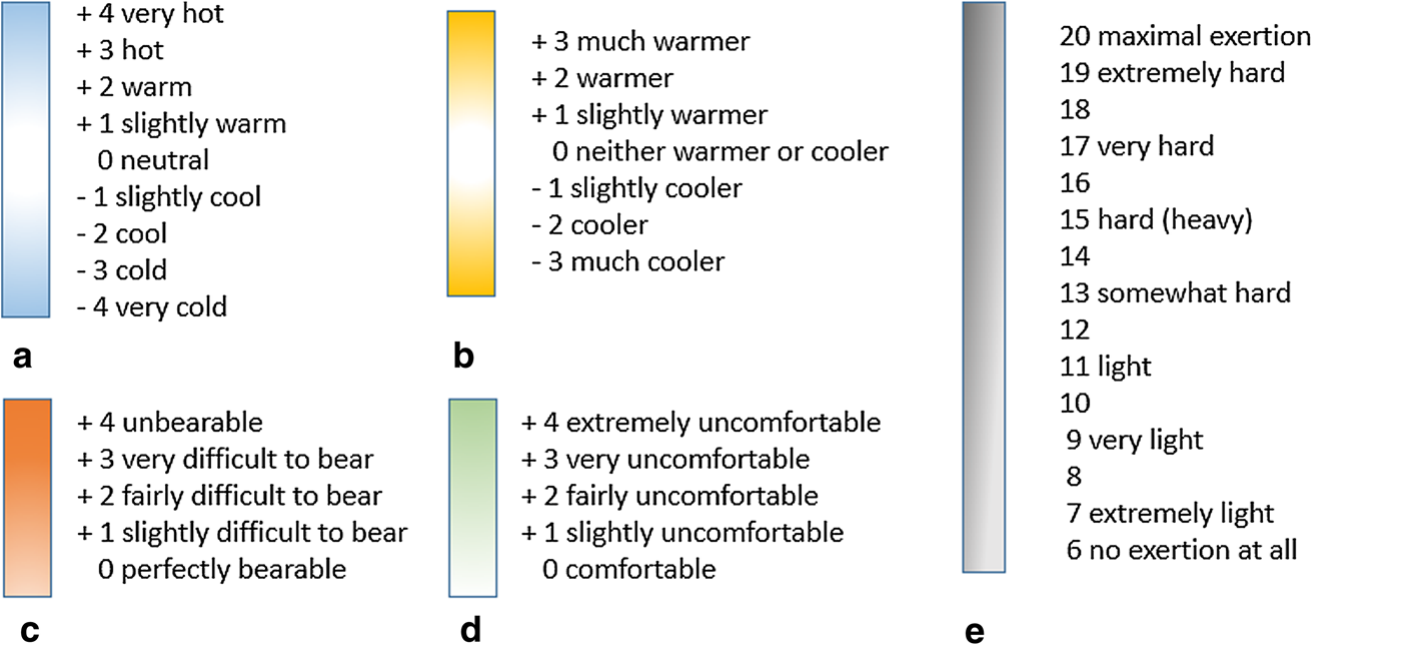
Subjective scales for: **a** Thermal perceptual, **b** personal thermal preference, **c** thermal tolerance, **d** affective evaluation, **e** RPE

Thermal sensation assessment was designed according to ISO-10551(ISO 1995), including thermal perceptual evaluation (7 degrees, from very cold to very hot), affective evaluation (5 degrees, from comfortable to very uncomfortable), personal thermal preference (7 degrees, from much cooler to much warmer), personal thermal acceptance (2 degrees, generally acceptable, general unacceptable), and personal tolerance (5 degrees, from perfectly tolerable to intolerable). Also, Borg rating of perceived exertion (RPE) (Borg 1990) was applied to determine the exercise intensity levels (6–20, from no exertion at all to maximal exertion). The detailed scales are shown in Fig. 2.

## Results

### Core temperature

**Fig. 3:**
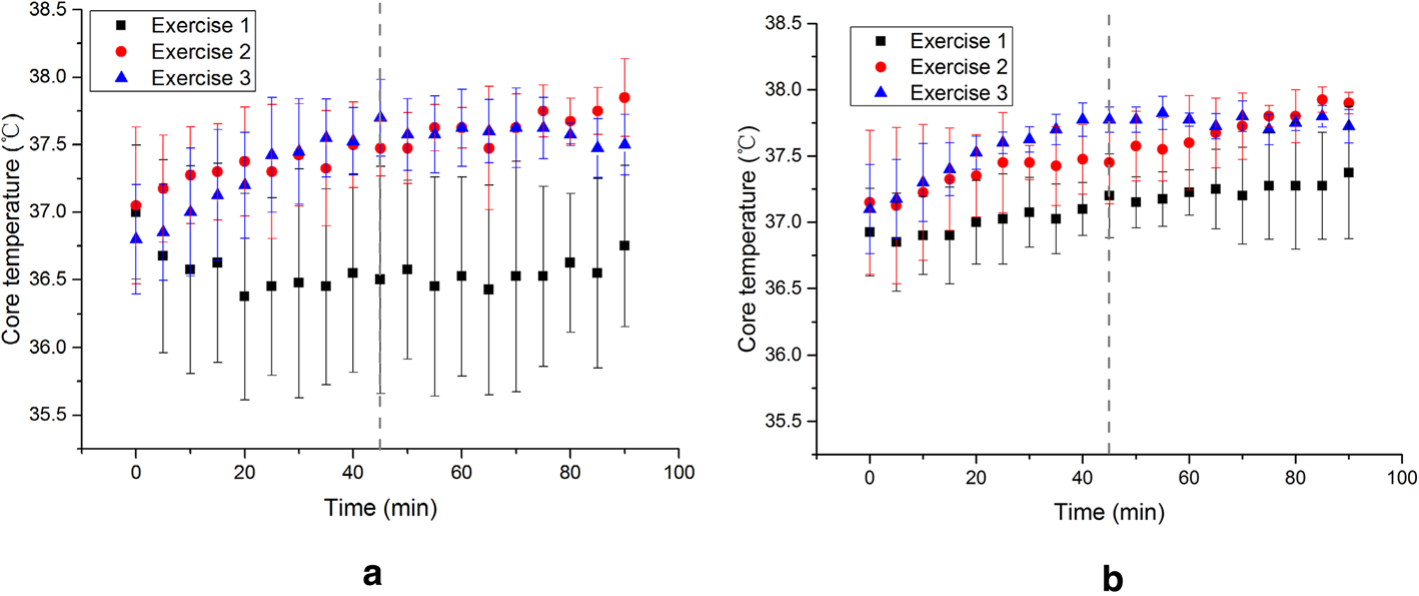
Rectal temperatures and their standard deviations in 3 exercises under different environment conditions. **a** 26 °C, 45–55 % RH, **b** 31 °C, 45–55 % RH

Rectal temperature served as an indication of core temperature (Schlader et al. 2009), which was measured in 3 exercises under different environment conditions as shown in Fig. 3.

At 26 °C, the rectal temperatures in Exercise 1 fluctuate little during the whole process of 90 min for each individuals, of which the difference between the maximum and the minimum is 0.6 °C. However, the rectal temperature of individuals in the resting state varies largely, of which the mean standard deviation is 0.72 °C. The rectal temperatures of Exercises 2 and 3 are 0.92 and 0.85 °C higher than that of Exercise 1 in average. The individual difference in Exercises 2 and 3 was much lower, and the mean standard deviations are 0.32 and 0.31 °C respectively. During Exercise 2, the core temperature keeps increasing along with time. But in Exercise 3, there is an obvious increase only in the first stage with the 6 km/h walking velocity while the core temperature in the second stage maintains at about 37.58 °C.

When the ambient temperature goes up to 31 °C, the individual differences of 3 exercises decrease, of which the mean standard deviations are 0.32, 0.30 and 0.14 °C respectively. Different with the condition of 26 °C, the rectal temperature in Exercise 1 runs slowly up about 0.53 °C from 36.85 to 37.38 °C during the 90 min. And the temperature of Exercise 2 and Exercise 3 are 0.41 and 0.52 °C higher than that of Exercise 1 in average (37.12 °C). With the moderate exercise intensity (3 km/h) in Exercise 2, the rectal temperature goes up and maintains at about 37.45 °C from 30 to 45 min. When the activity intensity rises to 6 km/h, the rectal temperature keeps rise a little quickly (0.125 °C/5 min compared to 0.1 °C/5 min). As for Exercise 3, the rectal temperature reaches a relative steady state at about 40 min, and there is only a slightly fall (about 0.12 °C) with the reduced intensity.

The core temperature of six groups starts at about 37 °C (±0.13 °C).In Exercise 2 and 3, the core temperatures are higher than that in Exercise 1 under both environmental conditions, and the standard deviation under condition of 31 °C, 45–55 % RH is much smaller than that of 26 °C, 45–55 % RH. The maximum rectal temperature growth rate of Exercise 3 is much higher than that of Exercise 2 in the first 45 min. And at 31 °C, 45–55 % RH, it (0.225 °C/5 min) is higher than that at 26 °C, 45–55 % RH (0.125 °C/5 min).

### Skin temperature

#### Local skin temperature

**Fig. 4:**
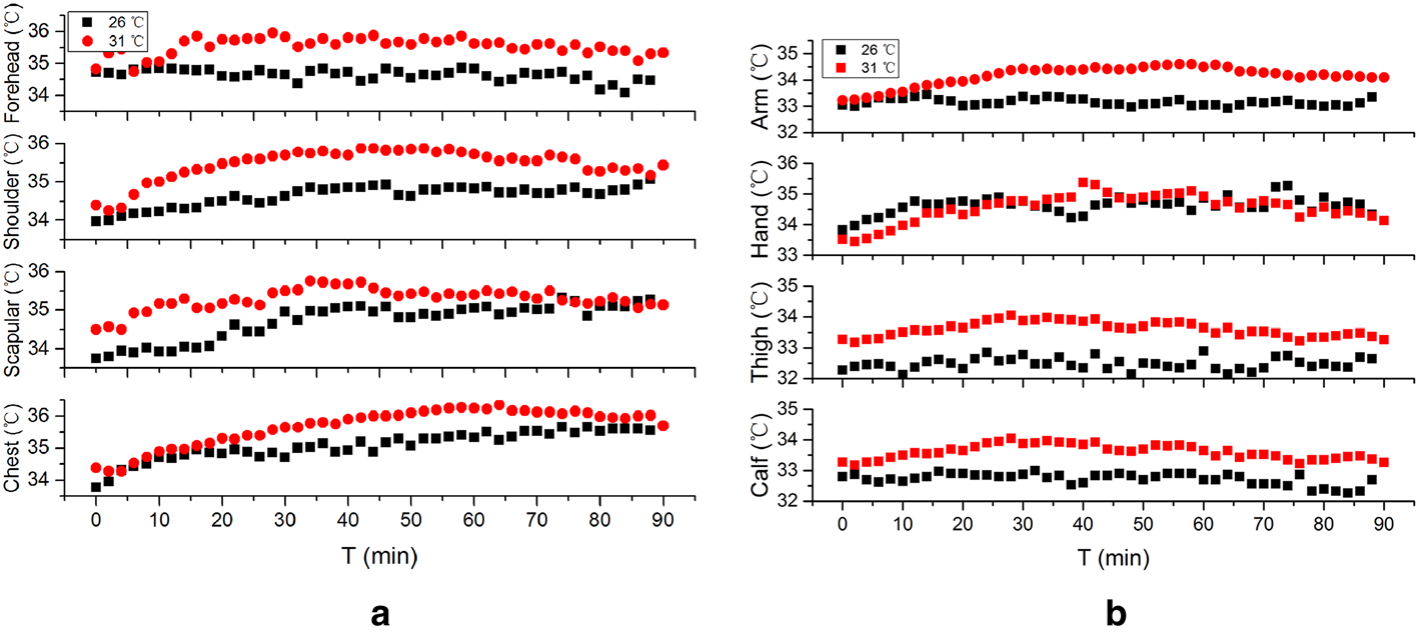
Comparison of local skin temperature at 26 °C, 45–55 % RH and 31 °C, 45–55 % RH in Exercise 1. **a** Skin temperature of trunk, **b** skin temperature of limbs

**Fig. 5:**
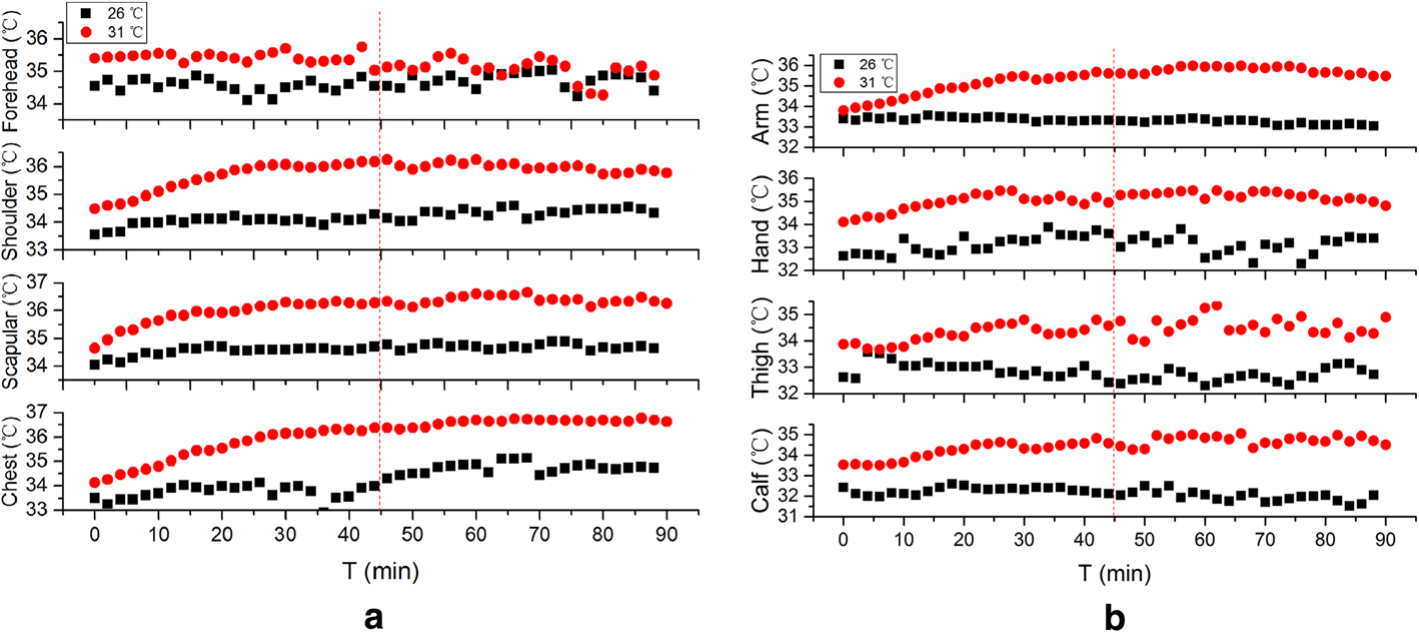
Comparison of local skin temperature at 26 °C, 45–55 % RH and 31 °C, 45–55 % RH in Exercise 2. **a** Skin temperature of trunk, **b** skin temperature of limbs

**Fig. 6:**
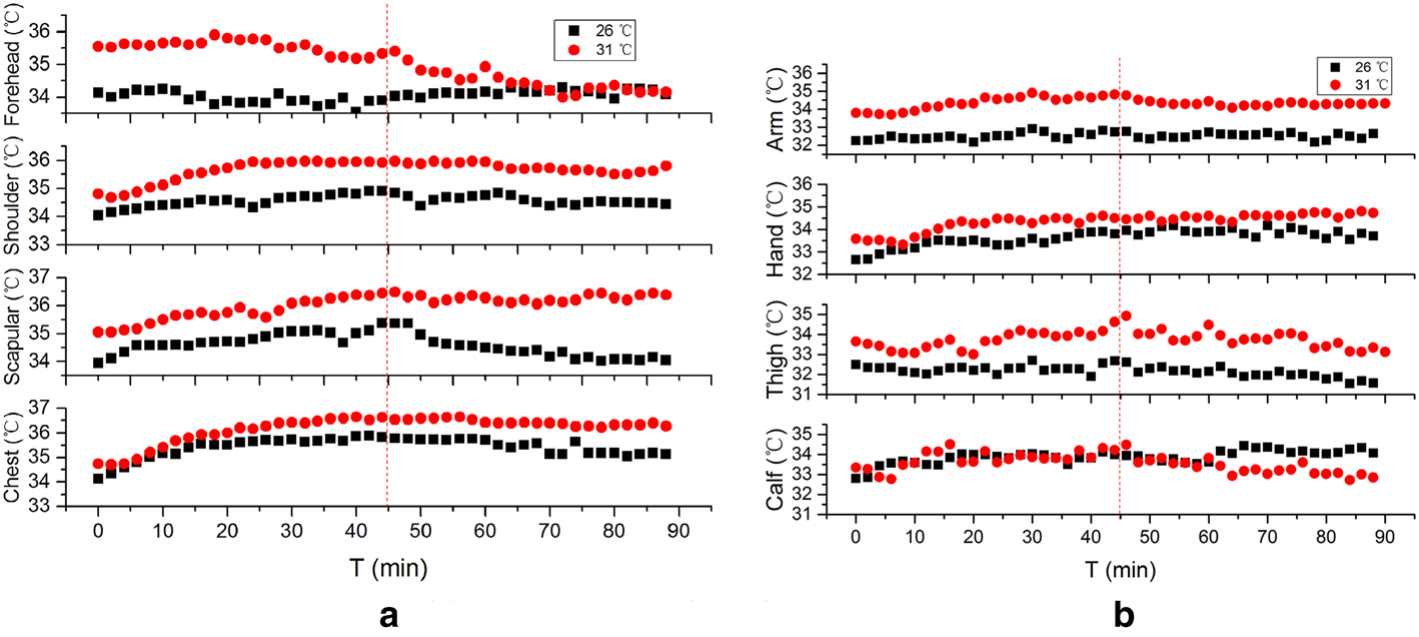
Comparison of local skin temperature at 26 °C, 45–55 % RH and 31 °C, 45–55 % RH Exercise 3. **a** Skin temperature of trunk, **b** skin temperature of limbs

Figures 4, 5 and 6 illustrate the subject local skin temperatures during the 3 exercises under different environment conditions.

As can be seen from the figures, it is clear that the local skin temperatures are higher at 31 °C, 45–55 % RH than that at 26 °C, 45–55 % RH in most cases. Among eight points measured, temperature interval of trunk (including forehead, chest, shoulder and scapular) are 35–35.4, 34.9–35.4 and 34.5–35.8 °C in Exercise 1, 2 and 3, which are higher than that of limbs (including arm, hand, thigh and calf) in each exercise (33–34.5, 33.2–34.4 and 32.9–34 °C) respectively .

**Fig. 7:**
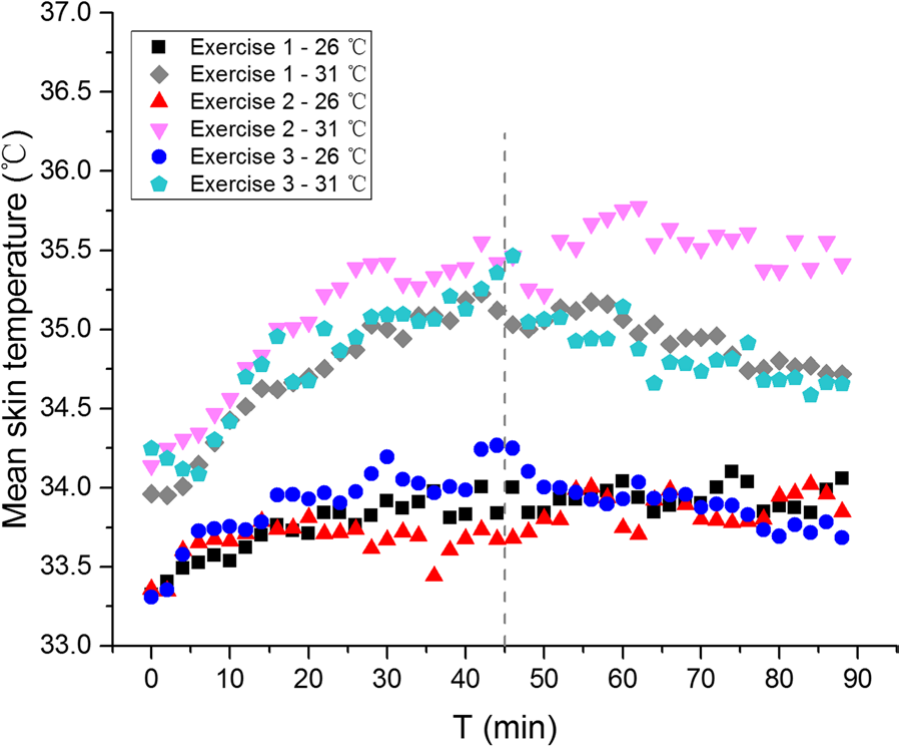
Mean skin temperature of 3 exercises at 26 and 31 °C

The forehead temperature of Exercise 1 keeps a relative balanced state. The average temperatures and deviations in each condition are 34.6 ± 0.2 and 35.5 ± 0.3 °C. Despite the environment condition, forehead temperatures finally become the same in Exercises 2 (about 34.8 °C) and 3 (about 34.2 °C). The temperatures of shoulder, scapular, chest and arm show the same tendency in Exercise 1: increase a little and then becoming unanimous with that in the other environment. However, along with the time, these temperatures of Exercise 2 rise quickly for about 30 min, and then the trend goes towards stable. Further increase of exercise intensity has little influence on the growth rate. As is shown in Fig. 7, high exercise intensity on the first stage of Exercise 3 minimizes the influence of ambient temperatures on local skin temperature except forehead compared to Fig. 6. It is obvious especially for hand and calf, of which mean difference values between comfortable and hot environment are 0.71 ± 0.26 and 0.54 ± 0.44 °C.

### Mean skin temperature

Mean skin temperature was weighted using the following 8 point formula (ISO 2004; Thornley et al. 2003):

1 $${\text{T}}_{\text{sk}} = 0.07{\text{T}}_{\text{forehead}} + 0.07{\text{T}}_{\text{shoulder}} + 0.175{\text{T}}_{\text{scapula}} + 0.175{\text{T}}_{\text{chest}} + 0.07{\text{T}}_{\text{arm}} + 0.05{\text{T}}_{\text{hand}} + 0.19{\text{T}}_{\text{thigh}} + 0.2{\text{T}}_{\text{calf}}$$

Under the comfortable environment condition on the first stage, the mean skin temperature of Exercise 2 is slightly lower than that of the Exercise 1, while that of Exercise 3 is slightly higher than static state. The mean skin temperature of 3 exercises reaches agreement to about 33.8 °C after 50 min exercise.

In the hot environment, there is a similar tendency of mean skin temperatures between of Exercise 1 and 3. The temperature increased to 35.3 °C in 45 °C min, and then decreased to 34.7 °C at last. However the mean skin temperature in Exercise 2 shows the same ‘increase-steady pattern’ in both two stages. It keeps increasing in 30 min to 35.4 °C and there is another increase to 35.8 °C during 50–60 min.

### Subjective judgment

**Fig. 8:**
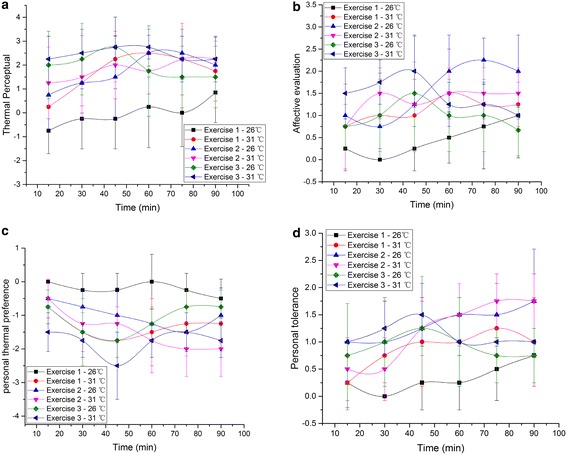
Subjective sensation during the experiment. **a** Thermal perceptual, **b** affective evaluation, **c** personal thermal preference, **d** personal tolerance

Subjective sensation data collected from the surveys is averaged and standard deviations are labeled (Fig. 8). All subjects accepted the two climate conditions in 3 exercises.

During Exercise 1, subjects feel the environment condition of 26 °C and 50 % RH neither too warm or cool (thermal perceptual ranging from −0.75 to 0.85), and no tendency is shown to change the environmental condition (thermal preference fluctuating from 0 to −0.5). Sitting at ease under this condition, subjects felt bearable (thermal tolerance ranging from 0 to 0.75) during the whole process, but slightly uncomfortable in the end (affective evaluation rose up to 1 at 90 min). Under the hot environment condition, subjects felt slightly warm. As the time went by, they felt it warmer (thermal perceptual ranging from 1.25 to 2.5), and slightly difficult to bear (thermal tolerance ranging from 0 to 0.75). But in the end, the uncomfortableness and their desire to get the environment cool lightened (thermal affective evaluation decreasing from 1.5 to 1.25, thermal preference increasing from −1.5 to −1.25).

At the first stage of Exercise 2, subjects felt slightly warm at 26 °C and warm at 30 °C (thermal perceptual increasing from 0.75 to 1.5 and from 1.25 to 2 respectively). And when the intensity increased to 6 km/h, subjects felt warm under both condition. But there is a fall (from 2.5 to 2) at this stage in thermal perceptual at 26 °C, while thermal perceptual kept rising from 1.75 to 2.25 at 31 °C. At the same time, subjects preferred to be slightly cooler but felt fairly uncomfortable at 26 °C, while they preferred to be cooler but felt slightly uncomfortable at 31 °C (thermal preference and affective evaluation were −1 and 2, −2 and 1.5 respectively). Thermal tolerance rose to 1.75 in the end, which meant that subjects felt slightly unbearable under both condition.

Subjects shared the same thermal perceptual sensation at the first stage of Exercise 3 under both environmental condition. They felt warm at start and nearly hot at 45 min (thermal perceptual increasing to 2.75). With the decrease of walking intensity, thermal perceptual fell to slightly warm (1.5) at 26 °C, and warm (2) at 31 °C. At 26 °C, subjects preferred the environment to be slightly cooler with thermal preference ranging from −0.75 to −1.25. However, thermal preference at 31 °C decreased from −1.5 to −2.5 during first 45 min and then recovered to −1.75, which meant subjects preference changing from slightly cooler to cooler and then to slightly cooler. At 26 °C, subjects felt slightly uncomfortable (thermal affective evaluation ranging from 0.75 to 1.5) and unbearable (thermal tolerance ranging from 0.75 to 1.25 during the first stage, and the uncomfortableness and unbearableness were lightened in the end with a fall to 0.67 and 0.75 respectively.

## Discussion

### Core temperature

Activity and high temperature narrows individual discrepancy on subject core temperature. In Fig. 3, it is clear that the standard deviation of core temperature of resting state is higher than the other 2 exercises under each conditions. And the standard deviation at 31 °C is lower compared to that at 26 °C.

A higher intensity increasement would bring a higher core temperature growth rate. This can be concluded from the slope comparison at the first stage of Exercise 2 and Exercise 3. With a higher velocity of Exercise 3, the slope is much larger than that of Exercise 2.

Exercise intensity of 0 km/h does not indicate the same stable state inside the human body because the core temperature changes according to the ambient environment (i.e. increasing at 31 °C, and decreasing at 26 °C). Compared to resting state (Exercise 1) in Fig. 3a, 5 °C increasement of ambient temperature broke the origin balance between human body and the environment. If given sufficient time, it can be predicted that the core temperature will finally reach a stable level.

A certain intensity of exercise may help subjects regain the new balance quickly under different climate condition. An obvious stable state is shown in Exercise 2 in about 30 min (Fig. 3b).

Sequence of velocity during the exercise plays a role in the final core temperature. On the premise of the same walking distance and environmental conditions, core temperature in the end in Exercise 3 (6 km/h in the first 45 min then 3 km/h in the left 45 min) is lower than that in Exercise 2 (3 km/h in the first 45 min then 6 km/h in the left 45 min). With the decrease of walking velocity in the second stage of Exercise 3, the core temperature decrease slightly (at 26 °C), or maintains relative stable (at 31 °C).

### Skin temperature

The ANOVA results of local skin temperatures and mean skin temperature are shown in Table 3. For the forehead, scapular, chest, arm, hand, thigh and calf, the mean square between groups is greater than that within groups. All the sig. value is less than 0.001, which indicated that the tested data is of significance.

**Table 3:** ANOVA for localized skin temperature and mean skin temperature

	Sum of square	df	Mean square	F	Sig.
Forehead					
Between groups	63.655	5	12.731	115.457	<0.001
Within groups	29.110	264	0.110		
Total	92.765	269			
Scapular					
Between groups	111.579	5	22.316	161.293	<0.001
Within groups	36.526	264	0.138		
Total	148.105	269			
Shoulder					
Between groups	102.097	5	20.419	179.762	<0.001
Within groups	29.988	264	0.114		
Total	132.086	269			
Chest					
Between groups	118.116	5	23.623	74.326	<0.001
Within groups	83.907	264	0.318		
Total	202.023	269			
Arm					
Between groups	228.973	5	45.795	405.167	<0.001
Within groups	29.839	264	0.113		
Total	258.812	269			
Hand					
Between groups	116.293	5	23.259	166.455	<0.001
Within groups	36.888	264	0.140		
Total	153.181	269			
Thigh					
Between groups	163.793	5	32.759	342.686	<0.001
Within groups	25.237	264	0.096		
Total	189.029	269			
Calf					
Between groups	155.066	5	31.013	269.853	<0.001
Within groups	30.340	264	0.115		
Total	185.406	269			
Mean skin					
Between groups	93.844	5	18.769	234.814	<0.001
Within groups	21.102	264	0.080		
Total	114.945	269			

As can be seen from Figs. 4, 5 and 6, the temperature of trunk is higher than that of limbs. Wearing a vest-style SRBA helps to prevent the heat produced by basic metabolism and exercise dissipating, which promote the increase of local temperature.

There is no explicit relationship between the local skin temperature and the exercise intensity. As is shown Fig. 5, during the first stage, constant increase of local temperature means that heat dissipation from Exercise 2 at 31 °C is lower than the heat production. This trend is weakened when the exercise intensity is higher.

Heat dissipation and heat production from exercise play important roles in the human skin temperature change. When the exercise intensity increases, the metabolic rate increases too. So more heat is produced via human body. Perspiration evaporation is an efficient way to take out the body heat, thus adjust human body surface temperature. But which one is more important depends both on exercise intensity and environment conditions. Compared the mean skin temperature on the first stage of 3 exercise at 26 °C on Fig. 7, skin temperature of Exercise 3 is higher than Exercise 1, which means heat production of 6 km/h is much more than heat dissipation. But for Exercise 2, the skin temperature is lower than Exercise 1, which means more heat dissipate than produced. In this case, exercise intensity has a greater influence on mean skin temperature. However, mean skin temperature of Exercise 2 and 3 at 31 °C shows the opposite results, which indicates that environmental condition has obvious effect on the heat dissipation.

### Subjective judgement

From the analysis of thermal sensations, it can be concluded that sequence of exercise intensity during the whole process plays a major part in the subjective thermal sensation. Richard de Dear (2011) suggested a concept of alliesthesia: *any external or environmental stimulus that has the precept of restoring the regulated variable within the milieu interieur to its set point will be perceived as pleasant (positive alliesthesia), while any environmental stimulus that will further displace the error between the regulated variable and its set point will be perceived as distinctly unpleasant, or even noxious in more extreme cases (negative alliesthesia)*. In our research, although the walking distance is the same, Exercise 3 makes the subjects more tolerable to the environment with the velocity decrease from 6 to 3 km/h in the end, and their desire to lower the temperature is less strong than Exercise 2. The decrease of the velocity in Exercise 3 is a pleasant stimuli thus leads to the positive alliesthesia, while the increase of the velocity in Exercise 2 is an unpleasant stimuli so the subjects want to avoid it. The change of exercise intensity is an external stimulus. And it plays the same role in this alliesthesia theory as the environmental stimulus. Luo et al. (2016) once concluded that if personal control approaches were utilized, the thermal discomforts might be reduced through a slightly improvement of thermal condition. The mechanism of the external or environmental stimulus affects the thermal comfort is still under development, so the occurrence and calculation methods of the alliesthesia is still unknown.

In order to discuss the relationship between physical parameters (rectal temperature and mean skin temperature) and subjective judgement (thermal sensations), regression analysis has been done as shown in Table 4. Positive or negative correlations are found, as shown in Table 4. The thermal perceptual, affective evaluation, thermal tolerance and rating of perceived exertion all present strongly positive correlations to the rectal temperature with Pearson’s r greater than 0.75. But no such obvious relationship was shown with mean skin temperature, for which the related coefficients are less than 0.5. The subjective parameter of Personal thermal preference has a negative correlations with both rectal temperature and mean skin temperature with Pearson’s r equal to −0.77505 and −0.67947 respectively. So unary liner regression model is used to describe the relationship:

2 $${\text{y}} = {\text{a}} + {\text{bx}}$$

**Fig. 9:**
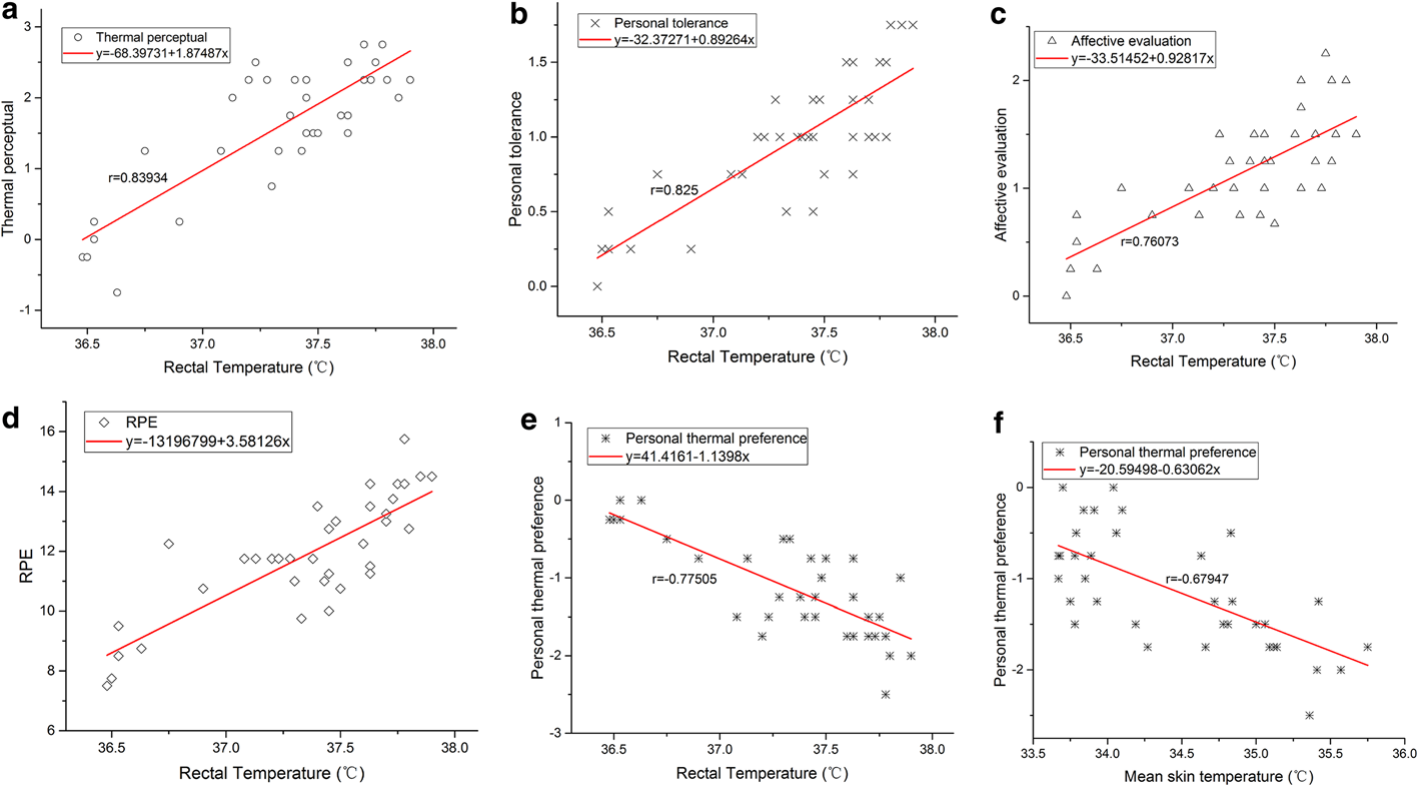
Regression equations between objective and subjective factors. **a** Rectal temperature and thermal perceptual. **b** rectal temperature and personal tolerance, **c** rectal temperature and affective evaluation, **d** rectal temperature and RPE, **e** rectal temperature and personal thermal preference, **f** mean skin temperature and personal thermal preference

Compared to the core temperature, skin temperature does not have such strong correlation to the subjective judgement. In this study, mean skin temperature only has a strongly significant negative correlation with thermal preference. Temperature in the body is more sensitive to the disturbance of the whole body thermal balance, and it can reflects the subjective thermal sensation and physical exertion. Significance test of the regression equation is conducted, as shown in Table 3. The sig. value is lower than 0.001, which indicates that regression equations is of statistical significance. Thus six regression equations were obtained as shown in Fig. 9.

**Table 4:** Regression analysis between the objective factors (core temperature and mean skin temperature) and the subjective factor (thermal sensations)

Independent factor	Dependent factor	Pearson’s r	Adj. R-square	a	b	Sig.
Core temperature	Thermal perceptual	0.83934	0.69581	−68.39731	1.87487	<0.001
	Affective evaluation	0.76073	0.56631	−33.51452	0.92817	<0.001
	Personal thermal preference	−0.77505	0.58895	41.4161	−1.1398	<0.001
	Thermal tolerance	0.825	0.680	−32.37271	0.89264	<0.001
	RPE	0.81989	0.66257	−131.96799	3.58126	<0.001
Mean skin temperature	Thermal perceptual	0.43316	0.16374	−19.43357	0.61063	0.008
	Affective evaluation	0.36825	0.11019	−8.62831	0.28356	0.028
	Personal thermal preference	−0.67947	0.44585	20.59498	−0.63062	<0.001
	Thermal tolerance	0.33164	0.08381	−6.85004	0.22662	0.049
	RPE	0.32059	0.07639	−20.9071	0.95038	0.057

Regression equation: $${\text{y}} = {\text{a }} + {\text{bx}}$$

Regression equations can be used to predict subjective sensation, if core temperature is given. For example, from the RPE curve (Fig. 9d), we can predict that when -rectal temperature reaches 37.6, subjects began to feel hard (15). And with the rectal temperature increase over 38, subjects would find the work heavy to carry on (17).

Objective physical parameters and subjective judgement would influence each other mutually. With the change of inner temperature, human body has thermal preference accordingly. Thermal preference then direct the thermoregulatory behavior of the body, including heat transfer with the environment. In this way, skin, acting as the media of the body’s direct interaction with the environment, plays an important role in the thermoregulatory behavior.

## Conclusions

The aim of this paper is to evaluate the impact of the exercise intensity on subjects when wearing the stab resistant body armor under comfortable and hot environment. Subjective and objective parameters are analyzed. Here are the conclusions:

1. Activity narrows individual discrepancy on core temperature, and a higher intensity increasement would bring a higher increasing rate.
2. A certain intensity of exercise may help subjects regain the new balance quickly under different climate condition.
3. Wearing a vest SRBA prevents the heat produced by basic metabolic and exercise dissipating, which is an important factor to the increase of local temperature.
4. Perspiration evaporation is a compensation to heat production from high intensity exercise. But the change of skin temperature depends both on exercise intensity and environment conditions.
5. Sequence of exercise intensity during the whole process plays a major part in core temperature and the subjective judgement. Within experimental conditions, decreasing of intensity at last stage which acted as the positive stimulus, makes the core temperature lower and the whole process much tolerable.
6. The core temperature is more sensitive to the disturbance (i.e. ambient temperature and exercise intensity) on the balance of the whole body, and it can reflects the subjective thermal sensation and physical exertion
7. Compared to core temperature, skin temperature do not have such strong correlation to subjective judgement. In this paper, mean skin temperature only has a strongly significant negative correlation with thermal preference.
8. Six unary liner regression models of body temperature and subjective sensations are gotten. Subjective an objective parameters can be predicted by each other roughly.

## Abbreviations

SRBA: stab resistant body armor
RH: relative humidity
SD: standard deviation
ANOVA: analysis of variance
